# Mass wasting susceptibility assessment of snow avalanches using machine learning models

**DOI:** 10.1038/s41598-020-75476-w

**Published:** 2020-10-27

**Authors:** Bahram Choubin, Moslem Borji, Farzaneh Sajedi Hosseini, Amirhosein Mosavi, Adrienn A. Dineva

**Affiliations:** 1 Soil Conservation and Watershed Management Research Department, West Azarbaijan Agricultural and Natural Resources Research and Education Center, AREEO, Urmia, Iran; 2 Reclamation of Arid and Mountainous Regions Department, Faculty of Natural Resources, University of Tehran, Karaj, Iran; 3 Environmental Quality, Atmospheric Science and Climate Change Research Group, Ton Duc Thang University, Ho Chi Minh City, Vietnam; 4 Faculty of Environment and Labour Safety, Ton Duc Thang University, Ho Chi Minh City, Vietnam; 5 Institute of Research and Development, Duy Tan University, Da Nang, 550000 Vietnam; 6 Kalman Kando Faculty of Electrical Engineering, Obuda University, Budapest, Hungary

**Keywords:** Geomorphology, Environmental sciences, Hydrology, Natural hazards

## Abstract

Snow avalanche is among the most harmful natural hazards with major socioeconomic and environmental destruction in the cold and mountainous regions. The devastating propagation and accumulation of the snow avalanche debris and mass wasting of surface rocks and vegetation particles threaten human life, transportation networks, built environments, ecosystems, and water resources. Susceptibility assessment of snow avalanche hazardous areas is of utmost importance for mitigation and development of land-use policies. This research evaluates the performance of the well-known machine learning methods, i.e., generalized additive model (GAM), multivariate adaptive regression spline (MARS), boosted regression trees (BRT), and support vector machine (SVM), in modeling the mass wasting hazard induced by snow avalanches. The key features are identified by the recursive feature elimination (RFE) method and used for the model calibration. The results indicated a good performance of the modeling process (Accuracy > 0.88, Kappa > 0.76, Precision > 0.84, Recall > 0.86, and AUC > 0.89), which the SVM model highlighted superior performance than others. Sensitivity analysis demonstrated that the topographic position index (TPI) and distance to stream (DTS) were the most important variables which had more contribution in producing the susceptibility maps.

## Introduction

Snow avalanche is among the most destructive natural hazards in the cold and mountainous regions with devastating socioeconomic and environmental impacts^1–4^. The mobility, transportation, tourism, and the leisure industries of the snowy mountain regions are under the avalanche's uncertain threats. Damaging infrastructures, roads and railways obstruction, threatening human life and the built environments and settlements, and harming the water resources, ecosystems, and vegetations are associated with the propagation and deposition of snow avalanche debris^5–11^. The devastating propagation of the snow avalanches may also contribute to the mass wasting of surface rocks and vegetation particles transported along the way and accumulated together with the snow avalanche debris^12^. The mass wasting induced by snow avalanche and the deposition of such snow-rock-debris poses longer-lasting damages with more destructive effects^4,13,14^. The global snow avalanche regime is increasingly reported to be changing within the past decade^15–18^. The climate change is introduced as a significant contributor in raising the occurrence rate and irregularity and increasing the risk and devastation^19–22^. Therefore, more than ever, the accurate spatial hazard modeling and susceptibility mapping of the avalanche slopes and hazardous locations are seen crucial for risk management, planning efficient mitigation and adaptation practices, and territorial land-use policies.

Modeling the avalanche triggering mechanisms is complicated^23–26^. The complexity of avalanche models has been discussed in many studies^27–31^. The snowpack, meteorology, terrain, and slope characteristics are the predominant contributing factors initiating the avalanche movement and propagation, and the debris deposition^2,32,33^. Based on the interaction of these factors, the motion and run out of snow and eventually, the avalanche formation and propagation can be modeled^2,32,34–36^.

Various numerical methods for modeling the avalanche flow dynamics^37–41^, as well as statistical approaches for processing historical database information and climatological data sets^15,42–45^, have been proposed to predict the hazard susceptibility mapping. The models have been enhanced with the involvement of recent advanced technologies of geographic information systems (GIS), remote sensing (RS), Satellite image processing, and artificial intelligence (AI) methods and applications^2,29,46–49^. GIS is a powerful tool for the construction of terrain’s geographical database to support building accurate prediction and decision-making models with high precision for terrain visualization^50–54^. Also, RS contributes to data collection through remotely sensing the inaccessible mountainous areas, which are a real asset in replacing costly and slow ground data collection systems without disturbing the snow cover^2,29,55,56^.

Machine learning (ML) has recently delivered fascinating results in advancing accurate models for susceptibility mapping of geohazards, e.g., earthquakes, landslides, earth fissures, and rockfalls^57–62^. However, the application of machine learning methods in avalanche prediction has been minimal^63,64^. Moreover, limited attention has been devoted to the hazard susceptibility of the mass wasting induced by snow avalanches which are accumulated as avalanche debris. As a response to these research gaps, this research's contribution is to model the hazard susceptibility of mass wasting by the snow avalanches in the mountainous region of Alborz Province, Iran, using well-known techniques that have not been used in this field so far.

This manuscript is structured in four sections. Section two presents the details of the study area, data, and the ML and the feature selection methods used for snow avalanche debris modeling. The results and discussion are described in section three. Section four presents the conclusions and discusses the ML methods' future direction for prediction of the snow avalanche mass wasting.

## Data and methods

### Description of the study area

**Figure 1:**
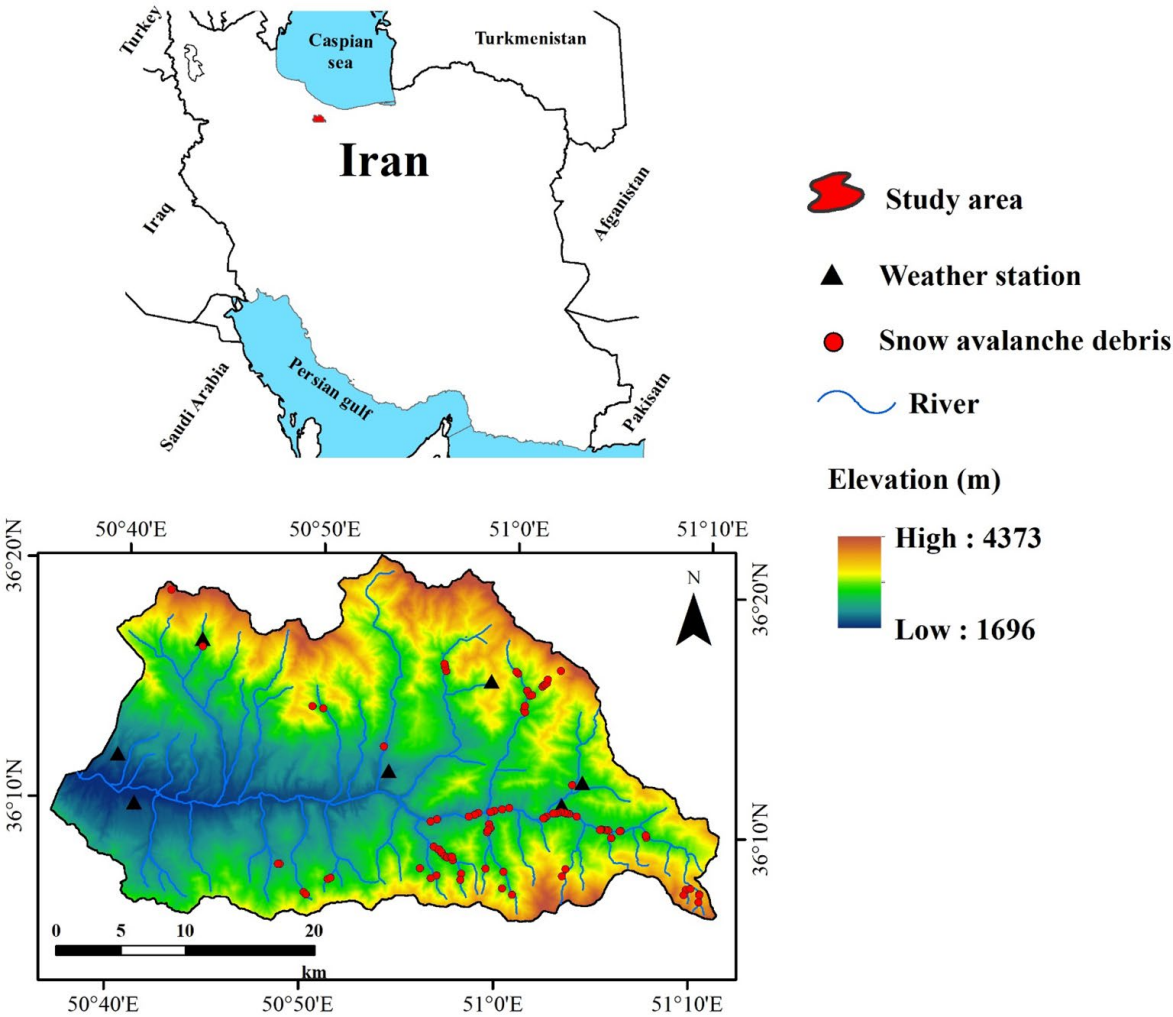
Location of the study area. The red circles denote locations of occurred snow avalanche debris. The black triangular indicate weather stations. The map was generated using ArcGIS Desktop 10.3, https://desktop.arcgis.com/en.

**Figure 2:**
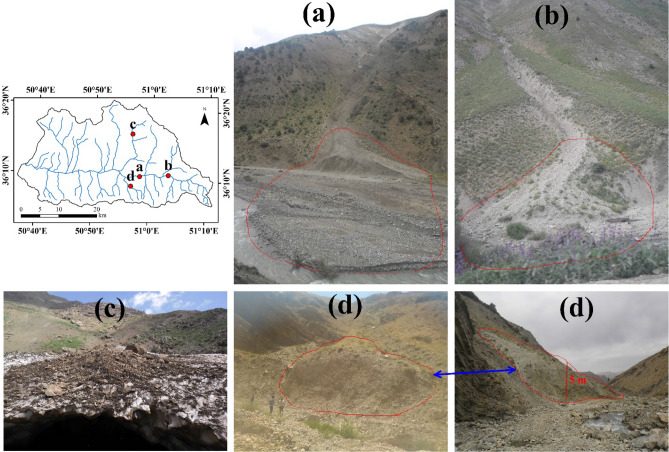
Some samples of snow avalanche debris which transferred the sediment into rivers in the region. The photographs were taken by Moslem Borji in 2019. The map was generated using ArcGIS Desktop 10.3, https://desktop.arcgis.com/en.

Taleghan watershed is located in the Alborz Province, Iran. It is extended from latitudes 36° 05′ to 36° 20′ N and longitudes 50° 36′ to 51° 11′ E with an area about 947 km^2^ (Fig. 1). The average annual temperature is about 11.4 °C and the average annual precipitation is about 520 mm. According to Hosseini^65^, most of the precipitation during the cold period (i.e., December to March) is as snow, which its ratio to annual precipitation varies between 33 and 51% during different years. The geology includes about 50 lithological strata, which the oldest is Precambrian, and the recent alluvial deposits are from the Pleistocene. This watershed’s lithology is mainly marly formations. The main soil orders of this region are Entisol and Inceptisol. Previous studies have shown sedimentation rates to be approximately 871 m^3^/km^2^/y; the preponderance of marly deposits accounts for a high erosion rate^66^. Considering how important the Taleghan dam is for metropolitan Tehran’s drinking water supply, the maintenance of the dam’s reservoir and reduction of sediment yield are crucial. Based on the long-term monitoring of river sediment, the annual sediment yield of the Taleghan watershed is about 10 tones/ha/y^67^. The Taleghan watershed is the upstream of two dams: the Taleghan (completed in 2006) and the Sefid-rood dam (completed in 1963), which both of them suffer from high sedimentation. One of the main sources of the watershed’s sediments is related to the snow avalanches debris, which deposits large volumes of sediment near the streams (Fig. 2). These deposits are exposed to being washed by the high flash floods. Therefore, it is important to know the hazardous snow avalanche debris areas to implement sediment-yield prevention initiatives.

### Inventory map of snow avalanche debris

Each snow avalanche consists of the starting zone, track, and runout zone^63^. After occurring a snow avalanche, the snow avalanche debris remains inside the snow runout (deposit) zone. As ice and snowmelt, these materials become visible (e.g., Fig. 2c). Most snow avalanches’ debris is very angular, enabling them to be differentiated from glacial, other drift-ice origins or rockfalls^68^. In this research, by considering the abovementioned conditions, a number of 98 locations of occurred snow avalanche debris were recorded based on the repeated field surveys (Fig. 1). In first, the probable locations of snow avalanche debris were selected based on the topographic maps and then based on the Google Earth. Then, through field surveys, the location of the avalanches containing sedimentary deposits was recorded. By observing the material accumulated in the snow at the end of the snow avalanche passages in the spring thaw period, the cause of this debris (i.e., snow avalanche) has been confirmed. Some samples of the recorded snow avalanche debris are shown in Fig. 2.

### Predictors used for snow avalanche debris modeling

**Figure 3:**
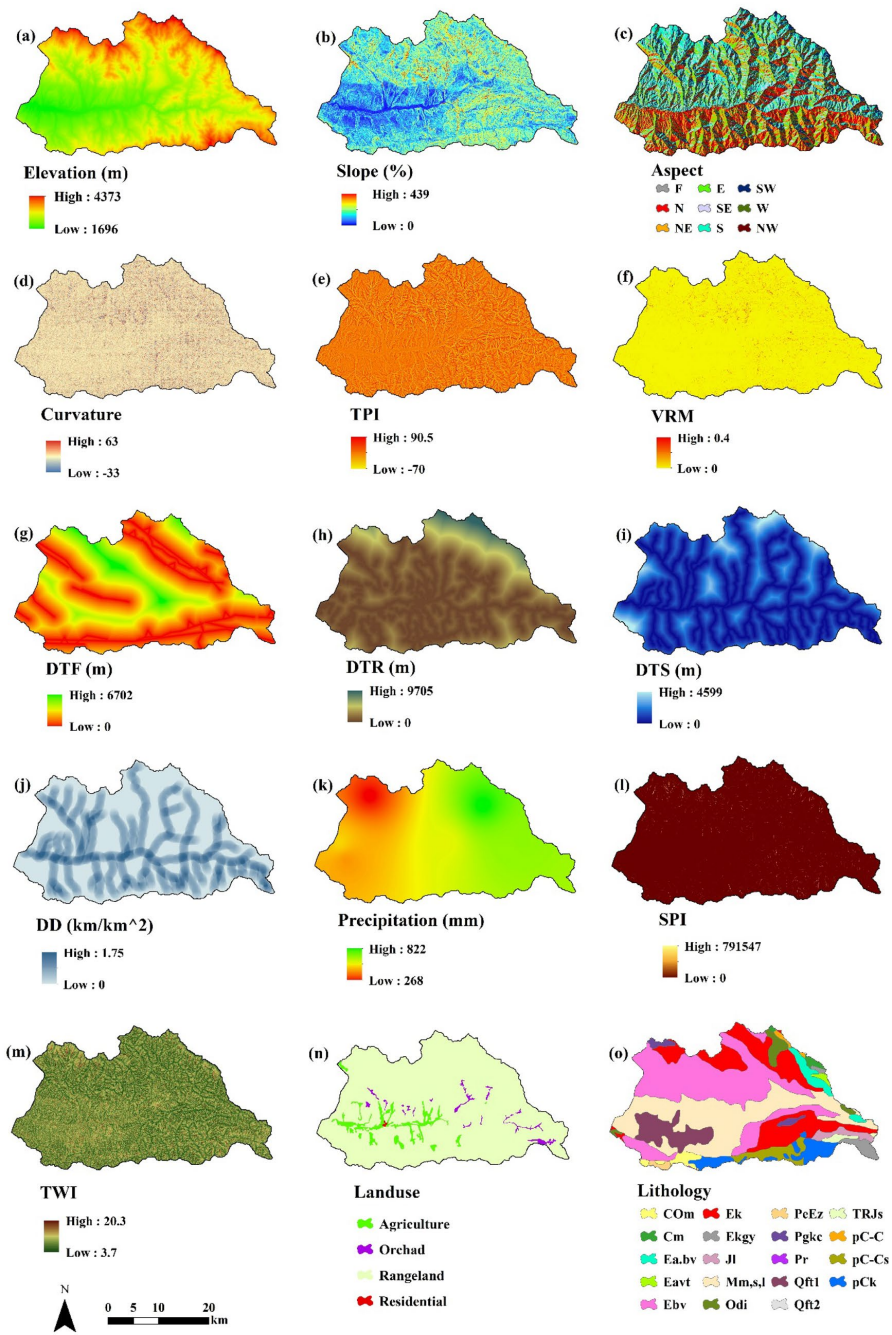
Snow avalanche debris conditioning factors: (**a**) elevation, (**b**) slope, (**c**) aspect, (**d**) curvature, (**e**) topographic position index (TPI), (**f**) vector ruggedness measure (VRM), (**g**) distance to fault (DTF), (**h**) distance to road (DTR), (**i**) distance to stream (DTS), (**j**) drainage density (DD), (**k**) precipitation, (**l**) stream power index (SPI), (**m**) topographic wetness index (TWI), (**n**) landuse, and (**o**) lithology. The maps were generated using ArcGIS Desktop 10.3, https://desktop.arcgis.com/en.

Snow avalanche debris can apply the considerable erosive forces on soils and may scrape away and entrain soil into the deposition zone. The chemical composition of the soil material entrained by the snow avalanche debris is changed and potentially contributing to form the landforms^69^. However, many factors affect the quantity of snow avalanche deposits and creating this natural hazard. These factors are including morphological features, climate, soil properties, vegetation cover, etc. In this study, for snow avalanche debris modeling, tried to consider the most related factors to the occurrence of snow avalanche debris:

- *Elevation:* Snow accumulation and melt are related to elevation (Fig. 3a), but the relationship between elevation and snow avalanche debris is more complicated than what is perceived in the watershed^70^. One of the most important factors that cause more deposition at the snow avalanche debris's runout zone is the pressure of snow. Snow pressure destroys rocky outcrops, and top layers of soil located on the crossing avalanches pass. Although elevation is not an inherent property of snow pressure, it affects the relative density of snow. The elevation factor contributes to increasing the relative density of snow, which increases the creep of snow fragments. Between the elevation of 1500 to 3000 m, for every 100 m of elevation, a two percent rise in snow pressure is calculated^71^. Thus, there is a direct relationship between the snow avalanche's elevation and the sediment yield^71^.
- *Slope:* High slopes (more than 50° or 120%) rarely contribute to snow avalanche debris, because they discharge continually during each new snowfall (Fig. 3b). Also, low slopes (less than 30° or 58%) no have the required potential (force) to create avalanches. However, slopes 30°–50° are more likely to the occurrence of avalanches. Due to the numerous factors that affect the snow avalanche debris, even with an equal gradient, the likelihood of avalanches in different places will not be the same^71^.
- *Aspect:* Geographic aspect (Fig. 3c) is of primary significance to avalanches. Different aspects have different influences on accumulating and melting of the snow because of the meteorological parameters, such as temperature, precipitation, wind velocity, and sunshine hours^63,64,72^.
- *Curvature*: Convex areas produce an increase in the rate of total motion of the snow cover downhill (a combination of creep and slide). In a suitable condition for avalanche releasing (presence of a stratum with low shear strength), the fracture will initially occur in these convex areas where there is tension force within the snow cover. So, the curvature map (Fig. 3d) can be an important layer in snow avalanche debris modeling.
- *Topographic Position Index (TPI):* The general concept and application of the TPI is to define and determine the boundary of landforms in an accurate and non-descriptive manner such as heights, steep slopes, flat areas, valleys, etc. using a DEM automatically and rapidly^73^. The TPI reflects the notion that the positive values of TPI represent areas that are higher than the surrounding points (hills), and the negative values represent regions with lower positions around them (valleys) (Fig. 3e). The values near zero indicate flat areas (where the gradient is close to zero) or areas with a steady slope^73^. This layer indicates valleys, streams, open slopes, mid-alluvial valleys, and u form valleys, which affect the occurrence of snow avalanche debris.
- *Vector Ruggedness Measure (VRM):* VRM (Fig. 3f) presents the roughness of the ground which, affects the movement of snow avalanche debris. For example, rocks and rock outcrops (i.e., irregular surfaces) may promote instability or stability of the snow avalanches^63^.
- *Distance to Fault (DTF):* The most important step in initiating the movement of thick snow fragments from the starting area is the formation of a weak layer fracture^74^. Many natural and anthropogenic factors such as load variation due to skiers, explosion, aircraft noise, and distance from active faults can cause these weak layer fractures (Fig. 3g).
- *Distance to Road (DTR):* This factor causes weak layers in the thick snow and increases the amount of debris deposition at the runout zone by moving vehicles on the road and generating heat and impact on local temperatures, as well as by the sound of beeps and vibrations (Fig. 3h).
- *Distance to Stream (DTS):* The snow avalanche deposits in the study area are directly related to the distance from the stream (Fig. 3i). Sediments accumulated along the river are transported by water flow and as the toe of the avalanche is emptied. So, the slope of the passage increased, then snow avalanche debris is increased.
- *Drainage Density (DD):* DD specifies the density of streams per unit ground, which is essential for occurring the snow avalanche debris occurrence^75^. Increasing the drainage density will create snow passage channels, and avalanche falls (Fig. 3j).
- *Precipitation:* Increasing the amount of precipitation increases the causes of the snow thickness in the area (Fig. 3k). Considering further essential factors to the avalanche formation, it is evidenced that snow-crossing discharge is increased by increasing precipitation. This would lead to increasing the debris.
- *Stream Power Index (SPI):* The SPI (Fig. 3l) indicates the erosive power of running water, directly affecting the slope toe erosion and stream incision^76^. So, it is the main factor that controls the slope erosion processes and snow avalanches.
- *Topographic Wetness Index (TWI):* The TWI (Fig. 3m), is a steady wetness index, which is commonly used to quantify topographic control on hydrological processes^75^. TWI is one of the most important factors that indicate the potential of runoff generation. In other words, the high values of TWI mean the high potential of runoff generation and vice versa. Therefore, in areas with high runoff, sediment deposits are transported downstream to different parts of the avalanche pass.
- *Land use:* Land use has a complex effect on the occurrence of avalanches and resulting deposits. For example, if the height of grass and bushes are higher than snow in rangelands, then the rangeland has a decreasing role in avalanche fall^71^. For extracting the land use map in this study, satellite data from the Sentinel-2 mission was chosen for two main reasons: (1) its relatively high 10 m spatial resolution, and (2) its radiometry includes three vegetation red edge bands. These two characteristics make the Sentinel-2 data appealing for land use extraction. According to the land use map, the rangelands include the region's highest area (Fig. 3n).
- *Lithology:* Lithology and rock units have an important role in the slope failure in mass movements such as avalanches, landslides, and debris flows^77^. Rock unit type is important in heat absorption and transfers to snow cover. Dark igneous rocks have the highest thermal absorption. These rocks can provide a suitable base for fracturing snow masses by absorbing heat and melting snow in the lower layers and creating pores in the snow masses. Conversely, light rocks such as lime and sandstone and igneous rocks absorb less heat and therefore control the movement of snow masses. Most rock units in areas with snow avalanche risk include andesite trace, basalt trace, basanite, andesite, agglomerate, tuff, and pyroclastic (Fig. 3o).

In this study, the ALOS PALSAR Digital Elevation Model (DEM) (https://vertex.daac.asf.alaska.edu/) with a pixel size of 12.5 m × 12.5 m was used to map the DEM derivatives (i.e., slope, aspect, curvature, TPI, VRM, SPI, and TWI). The spatial resolution of other input factors was resampled into an equal resolution of the DEM derivatives (i.e., 12.5 m × 12.5 m) using the Resample Tool in the ArcGIS environment. Also, the number of pixels and the extend of layers were controlled to be same for the spatial modeling.

### Multicollinearity analysis

Multicollinearity expresses the linear relationship between two or more independent variables. It is a data problem that may cause serious difficulty with the reliability of the estimates during the modeling process^78^. In this study, the Variance Inflation Factor (VIF) was used to assess the multicollinearity among the explanatory variables. Since the VIF values are low (VIF < 10) (Supplementary Table S1), there is not any multicollinearity among the predictor variables^59,79^.

### Feature selection using the recursive feature elimination (RFE)

The RFE procedure is generally a recursive process that ranks features based on the value of their importance. The method tends to eliminate weak features that are not important when synthesized with other features, and therefore, leads to a considerable decrease in the redundant variables^80^. The RFE method repeated many times and, in each time, the important features remained and the less important features are eliminated. The recursion is required because the relative significance of each feature can be essentially changed when assessing with a different subset of features in stepwise exclusion^81,82^. In this study, the RFE method was implemented using the k-fold (tenfold) cross-validation methodology by the Caret R package^83^.

### Susceptibility modeling of snow avalanche debris

Four machine learning (ML) models, including generalized additive model (GAM), multivariate adaptive regression spline (MARS), boosted regression trees (BRT), and support vector machine (SVM), were used to model the snow avalanche debris. Dependent data were the location of the snow avalanche debris (Fig. 1) and the independent data were the effective environmental factors identified by the RFE method. Locations of 98 snow avalanche debris, as well as the equal number of non-occurrence points, are converted to a binary scale (respectively 1 and 0) and considered as dependent data. The k-fold cross-validation methodology (k = 10) was used to model calibration in the R software environment using the ‘sdm’ package^84^. A ratio of 70% to 30% was considered respectively for calibrating and testing phases. Description of the applied ML models as follows:

The GAM model, developed by Hastie and Tibshirani^85^, blends the properties of the generalized linear model (GLM) with additive models. Indeed, this model is a statistical model in which the linear relationships between the dependent variable and independent variables are replaced by non-linear smooths^86^. On the contrary of the GLM, which the researcher needs to have proper knowledge of the correct functional form before the modeling, the GAM uses the additive approach in which the suitable functional form is selected based on the data^87^. To spatial modeling with the GAM model, as well as the ‘sdm’ package, the ‘mgcv’^88^ and ‘gam’^89^ R packages are required.

The MARS model, introduced by Friedman^90^, is a non-parametric statistical method which acts based on the divide and conquers strategy. This means that the calibration datasets are partitioned into separate splines (piecewise linear segments) of differing slopes (gradient)^91^. Indeed, it is known as an extension of linear models which automatically explores and models nonlinearities between the dependent and independent variables, without considering any assumptions about their relationships^92^. In this study, the ‘earth’^93^ package, as well as the ‘sdm’ package, was used to spatial modeling of snow avalanche debris by the MARS model.

The BRT model is a combination of boosting and decision tree methods. It frequently fits many decision trees to increase the model's accuracy, like the random forest (RF) model^94,95^. The model takes the random subsets, from the complete dataset with the same number of data points, for new trees that are built. According to the boosting method used in this model, the model continuously tries to improve its accuracy in each new tree which is built^96,97^. In this study, in addition to the ‘sdm’ package, the ‘gbm’^98^ package is required for spatial modeling using the BRT model.

The SVM model, introduced by Cortes and Vapnik^99^, is a non-parametric statistical monitoring method, which, to solve problems it uses the structural-risk minimization principle along with the dimension theory of Vapnik Chervonenk^100–102^. The mathematical function (kernel) transforms the data (inputs) into the required form and maps into a high dimensional feature space. The model creates a line (or a hyperplane) that splits the data into classes of 0 (non-occurrence) and 1 (occurrence). The SVM model with the kernel of radial basis function (RBF) was implemented using the ‘kernlab’^82^ R package, which is a dependent package in spatial modeling by the ‘sdm’ package.

### Modeling evaluation

Modeling results were evaluated using the several metrics which are used for evaluation of the dichotomous forecasts. These metrics are including the area under the curve (AUC) of receiver operator characteristic (ROC) plot, Accuracy (Eq. 1^103^), Kappa (Eq. 2^104^), Precision (Eq. 4^105^), and Recall (Eq. 5^105^) metrics which are calculated by the contingency table^106^:

1 $$\text{Accuracy } = \frac{\text{H} + \text{CN}}{\text{H+CN+M+CN}}$$

2 $${\text{Kappa}}\text{ } = \text{ }\frac{{\text{Acc}} \, - \, {\text{P}}_{\text{e}}}{\text{1 }- \, {\text{P}}_{\text{e}}}$$

3 $${\text{P}}_{{\rm e}} =  \frac{(\text{H } + \text{FA})(\text{H } + \text{ M})+ (\text{M } + \text{ CN})(\text{FA } + \text{CN})}{({\text{H}} + {\text{FA}} + {\text{M}} + {\text{CN}})^{2}}$$

4 $${\text{Precision}}\text{ } = \text{ }\frac{ \, \text{H }}{\text{H } + \text{ FA }}$$

5 $${\text{Recall}}\text{ } = \text{ }\frac{ \, \text{H }}{\text{H } + \text{ M }}$$

where H, FA, M, and CN are respectively the number of hits, false alarms, misses, and correct negatives^63,106^. ${\text{P}}_{\text{e}}$ represents the expected agreement between the modeled and forecasted values^104^. Variations of the evaluation metrics are between 0 and 1 which 1 indicates perfect perdition^107^.

## Results and discussion

### Feature selection results

Results of the recursive feature elimination (RFE) method indicated that the contribution of eight features, among 16, in snow avalanche debris modeling has better results (Accuracy = 0.963; Kappa = 0.926), according to the resampling performance using tenfold cross-validation method (Table 1). Variations of the accuracy with the different numbers of the features during the model runs are presented in Supplementary Figure S1. As can be seen, the mean accuracy (red plus in Supplementary Figure S1) with eight features is higher than the other number of the features. Therefore, based on the occurrence frequency of the features in the model (RFE) runs through the resampling process with a tenfold cross-validation method, eight variables with a higher occurrence frequency than others are selected as key features (Supplementary Figure S2). A higher occurrence frequency of a variable indicates its higher importance because the RFE is a backward select method^108^, which the lower important variables are removed from modeling and higher important variables are reminded^109^. Accordingly, variables of TPI, DTS, SPI, lithology, precipitation, TWI, DTR, and VRM with the contribution of 100%, 93%, 86%, 80%, 72%, 68%, 55%, and 53% in the model runs are selected as key features and used to snow avalanche debris modeling (Supplementary Figure S2).

**Table 1:** Resampling performance for different number of variables using the tenfold cross-validation method.

Variable	Accuracy	Kappa
1	0.867	0.737
2	0.928	0.856
3	0.942	0.883
4	0.956	0.912
5	0.949	0.898
6	0.956	0.912
7	0.956	0.912
8	0.963	0.926
9	0.956	0.912
10	0.942	0.883
11	0.953	0.916
12	0.956	0.912
13	0.956	0.912
14	0.949	0.898
15	0.956	0.912

Unlike the results of Mosavi et al.^64^ in snow avalanche susceptibility mapping, variables of slope and aspect in this study were eliminated during the feature selection. The reason is that, unlike the snow avalanche which consists of three zones^63^, the avalanche debris is located at the end of the snow avalanche passage. So, they mostly have a low slope and no aspect (i.e., flat) which cannot produce sufficient variance to be selected as important variables in snow avalanche debris modeling.

### Snow avalanche debris modeling

**Figure 4:**
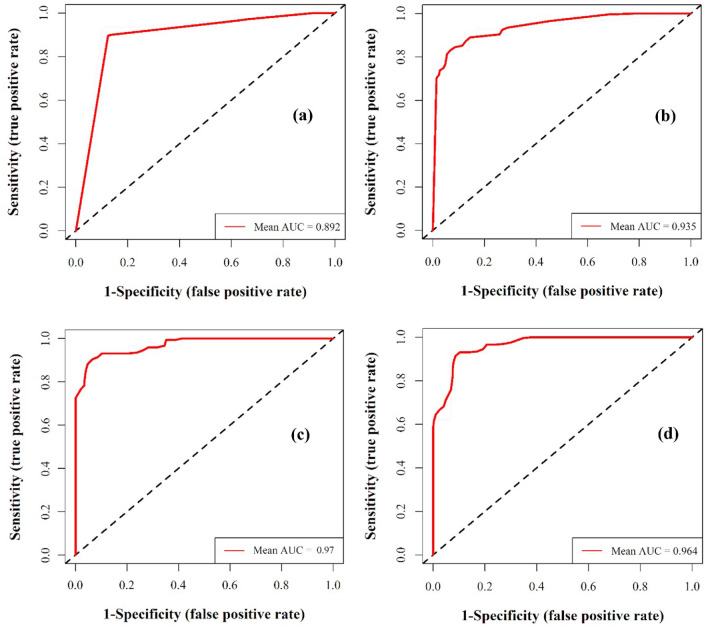
The area under curve (AUC) values: (**a**) GAM, (**b**) MARS, (**c**) BRT, and (**d**) SVM. Presented AUCs are mean AUC across tenfold cross-validation methodology.

Model calibration and validation were conducted using the key features identified by the RFE method. The AUC of ROC plots is presented in Fig. 4. As can be seen, according to the mean AUC across tenfold cross-validation methodology, the BRT and SVM models have close AUC values (respectively 0.970 and 0.964) and higher than the MARS (AUC = 0.935) and GAM (AUC = 0.892) models (Fig. 4).

For further understanding of the models’ performance, the Accuracy, Kappa, Precision, and Recall values for the testing dataset are calculated (Table 2). Results of the model evaluation indicated that the SVM model had a higher Accuracy, Kappa, Precision, and Recall values (respectively equal to 0.91, 0.83, 0.90, and 0.93) rather than other models (Table 2). Other models (i.e., BRT, MARS, and GAM) had the same performance given the Accuracy and Kappa values (respectively equal to 0.88 and 0.76), while the MARS model in view of Precision (equal to 0.89) and the BRT model in view of Recall were the best ones, after the SVM model (Table 2). However, the SVM model has different characteristics that make it successful in classification studies such as (i) applying kernel functions to solve the problems of linear separating classes resulting from multiple independent input variables^110,111^, (ii) maximizing the boundaries between classes for achieving an optimal separating hyperplane^110^, and (iii) excluding the outliers to avoid over-fitting of the model^111,112^.

**Table 2:** Snow avalanche debris modeling performance. A higher value of the statistics indicates a higher performance of the modeling.

Statistic	GAM	MARS	BRT	SVM
Accuracy	0.88	0.88	0.88	0.91
Kappa	0.76	0.76	0.76	0.83
Precision	0.87	0.89	0.84	0.90
Recall	0.90	0.86	0.93	0.93

### Spatial prediction of the snow avalanche debris susceptibility

After model calibration and evaluation, the spatial perdition of the snow avalanche debris susceptibility was done using the calibrated models and the pixels’ value of the predictors for the whole region. A natural break (Jenks) classification method was used to classify the predicted values into three low, moderate, and high classes. This method is based on the inherent classification in data which distinguishes classes based on the minimum difference within groups and the maximum difference between groups. Also, in other related studies such as snow avalanche hazard mapping (e.g., Choubin et al.^63^; Mosavi et al.^64^) this method has been used.

**Figure 5:**
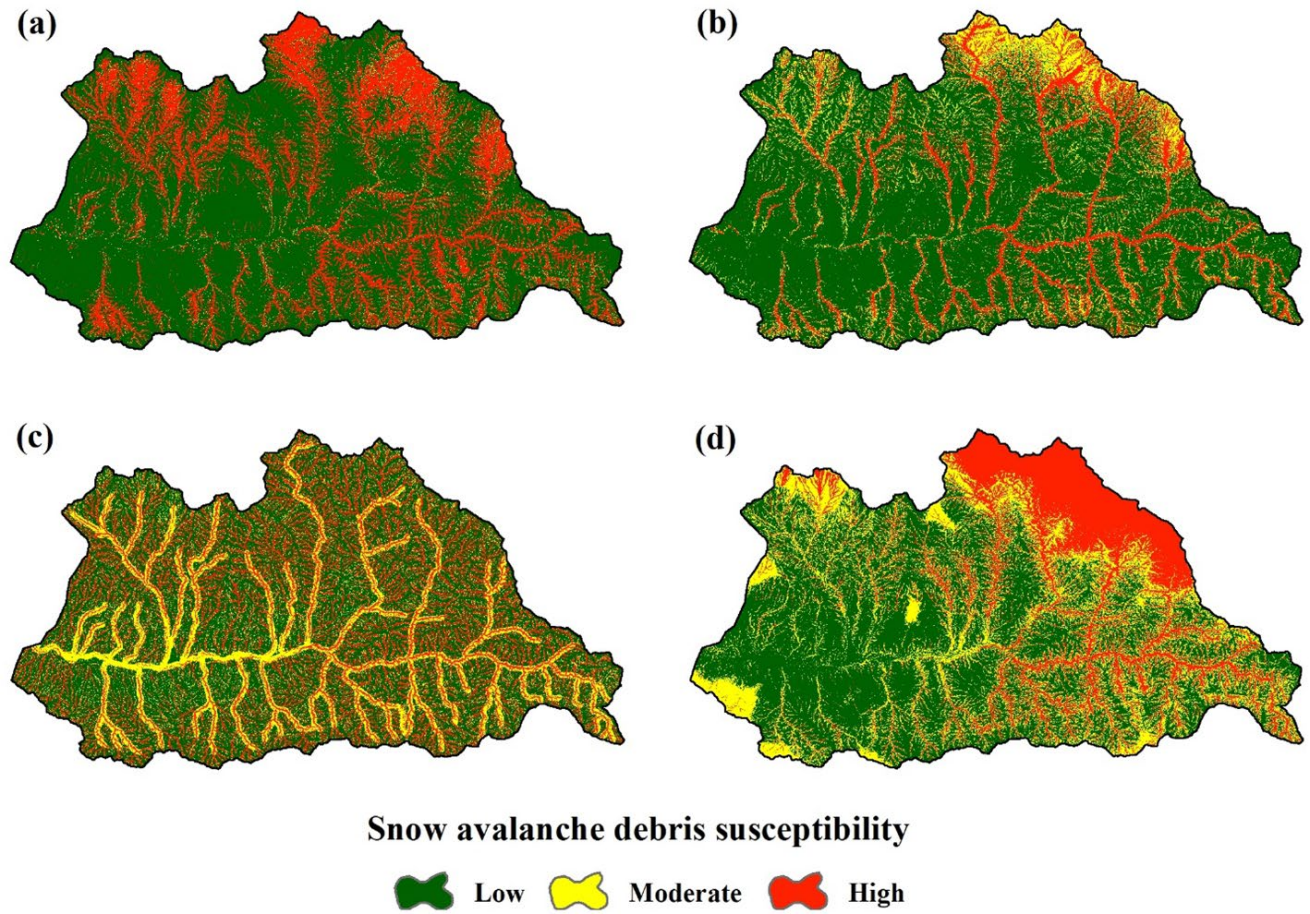
Snow avalanche debris susceptibility maps predicted by the GAM (**a**), MARS (**b**), BRT (**c**), and SVM (**d**). Low, moderate, and high susceptibility areas are shown in green, yellow, and red colors, respectively. The maps were generated using ArcGIS Desktop 10.3, https://desktop.arcgis.com/en.

The predicted susceptibility maps by the models with a pixel size of 12.5 m × 12.5 m are shown in Fig. 5. The susceptibility classes area (%) predicted by the models are presented in Table 3. The GAM and MARS models have the most area of the region in the moderate class (respectively 48.6% and 44.6% of the study area), while the most area of the region in the BRT and SVM models are related to the high susceptibility class (respectively 36.6% and 58.5% of the study area).

**Table 3:** The susceptibility classes area (%) predicted by the models.

Class	GAM	MARS	BRT	SVM
Low	16.5	25.5	35.7	12.1
Moderate	48.6	44.6	27.7	29.4
High	34.9	29.9	36.6	58.5

**Figure 6:**
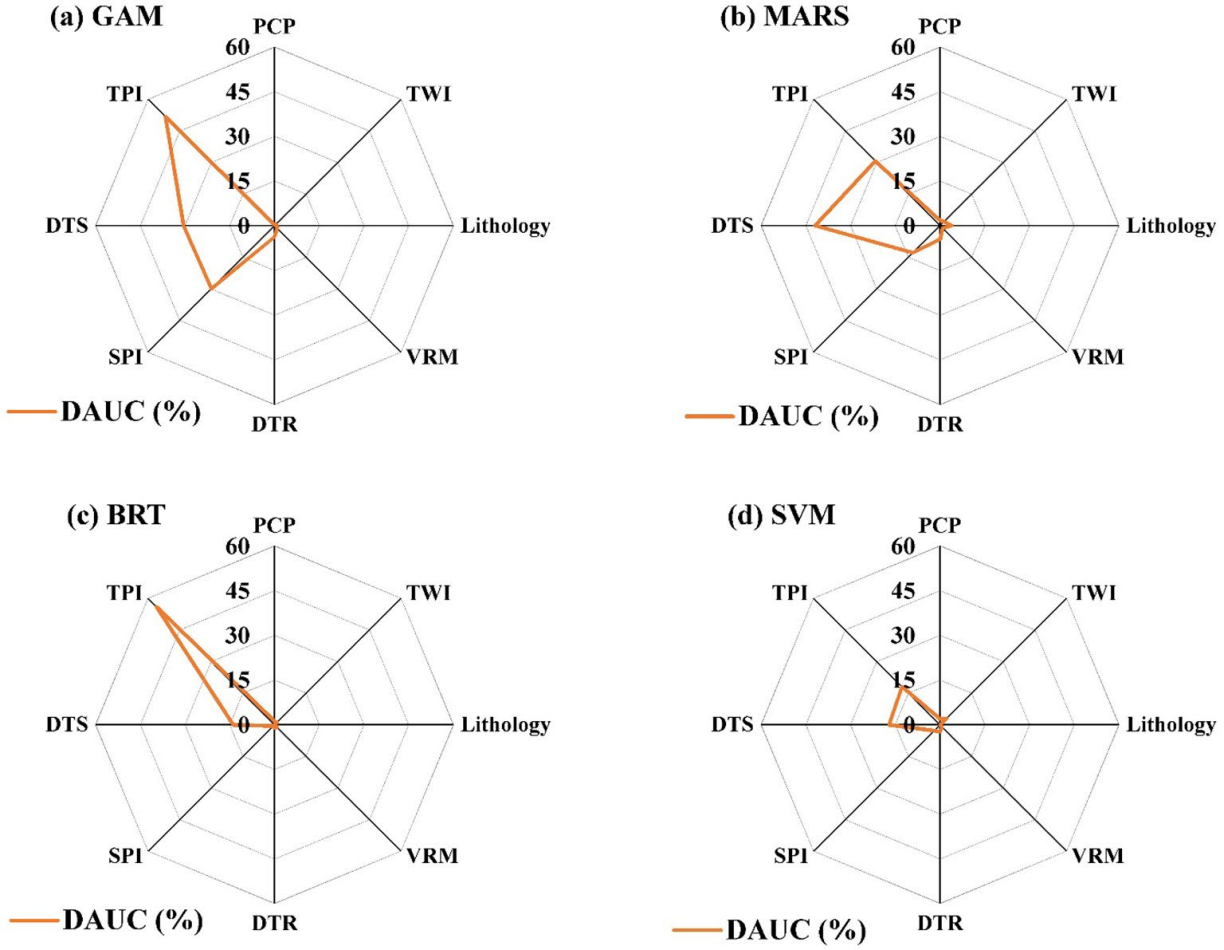
Importance of the variables based on the decrease in AUC (DAUC). A higher DAUC indicates a higher importance.

The results of susceptibility maps are different in each model (Fig. 5). The main reason refers to the different structures of the models which result in different outputs^107^. Also, this can be more clarified by the importance analysis of the variables according to the decrease in AUC (DAUC) values after excluding the parameters from the modeling process (Fig. 6). For the GAM model, variables of TPI (DAUC = 51.7%), DTS (DAUC = 30.5%), and SPI (DAUC = 29.9%) have more contribution to the modeling (Fig. 6) and the predicted map (Fig. 5a) is more match with them. In the MARS model, three main factors are respectively DTS (DAUC = 41.9%), TPI (DAUC = 30.7%), and SPI (DAUC = 12.8%). In this model, since the DTS is more important than the other variables, the susceptibility map (Fig. 5b) is more line with the DTS. Regarding the BRT model, the TPI (DAUC = 55.9%) is the main factor (Fig. 6). So, the high susceptible areas in this model (Fig. 5c) are matched with high values of TPI (i.e., heights) (Fig. 3). According to the SVM result, TPI and DTS are the most important variables with close importance (respectively DAUC equal to 17.1% and 18.1%) (Fig. 6). In this model, the high susceptibility areas are situated in mountainous regions of the northeast with high TPIs, that some glaciers of the Alam Kuh area are located in these areas. Also, the areas near to streams show a high susceptibility that this decreases from east to west (Fig. 5d). The reason for this can be because of decreasing the elevation and precipitation towards the west (Fig. 3).

Generally, the sensitivity analysis indicated that the most important variables were TPI and DTS with different importance in different models. Also, SPI was another variable that had a significant role in the GAM and MARS models (Fig. 6). Although limited attention has been devoted to the hazard susceptibility of the mass wasting induced by snow avalanche, in other nearby fields such as snow avalanche modeling, the importance of the TPI variable has been proved by Choubin et al.^63^ and Mosavi et al.^64^.

## Conclusions

Mass wasting induced by snow avalanche is among the major natural hazards in the cold and mountainous regions. Current research tried to model this natural hazard, for the first time, by considering the related environmental factors. Key features in this study were TPI, DTS, SPI, lithology, precipitation, TWI, DTR, and VRM which were identified by the recursive feature elimination (RFE) method (with an Accuracy equal to 0.963 and Kappa equal to 0.926). Modeling results with key predictors produced good results (Accuracy > 0.88 and AUC > 0.89). However, model comparison highlighted a superior performance of the SVM model (i.e., Accuracy = 0.91, Kappa = 0.83, Precision = 0.90, and Recall = 0.93) rather than the BRT, MARS, and GAM models. Results from the sensitivity analysis indicated that TPI and DTS variables were the most important factors that had more contribution in producing the susceptibility maps. Lack of the soil (or near-surface) temperature data in the study area was the main limitation of the research. Near-surface temperature can affect the avalanche movement which stimulates depleting the snow from hillsides and bringing the rock materials by itself into the runout zones (down the hillsides). Although this data is not available in the watershed area, its existence can help future studies in modeling the snow avalanche debris on the hillside scale. However, the maps produced by this study can help the watershed managers and land use policymakers to identify and protect the vulnerable areas.

## Supplementary information

Supplementary Information
