# Supplementary material for: Mass wasting susceptibility assessment of snow avalanches using machine learning models
Source: Sci Rep. 2020 Oct 27;10:18363. doi: 10.1038/s41598-020-75476-w (PMC7591884; doi:10.1038/s41598-020-75476-w)
Supplement: Supplementary file 1 — Supplementary Information [file 41598_2020_75476_MOESM1_ESM.pdf]

SUPPLEMENTARY MATERIALS FOR

Mass Wasting Susceptibility Assessment of Snow Avalanches Using Machine Learning Models

by

Authors: Bahram Choubin, Moslem Borji, Farzaneh Sajedi Hosseini, Amirhosein Mosavi, Adrienn A. Dineva

Corresponding authors: Amirhosein Mosavi (amirhosein.mosavi@tdtu.edu.vn) and Adrienn A. Dineva (adrienndineva@duytan.edu.vn)

This PDF includes:

Table S1

Figure S1

Figure S2

Table S1. Multicollinearity analysis

Variable	VIF	Variable	VIF
Aspect	1.02	Lithology	1.20
Curvature	1.84	Precipitation	1.23
Drainage density	2.43	Slope	1.75
Distance to fault	1.11	Stream power index	1.50
Distance to road	1.90	Topographic position index	2.71
Distance to stream	2.30	Topographic wetness index	2.83
Elevation	2.26	Vector ruggedness measure	1.08
Landuse	1.17	-	-

Note: VIF = Variance Inflation Factor

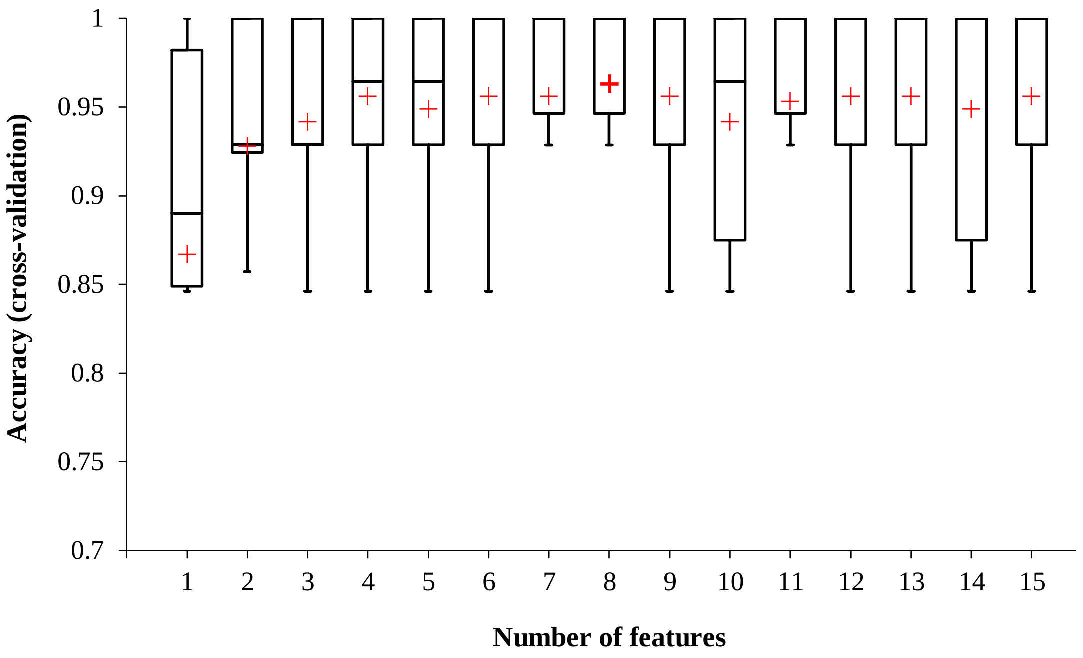

Figure S1. Variations of recursive feature elimination (RFE) performance with a different number of features.

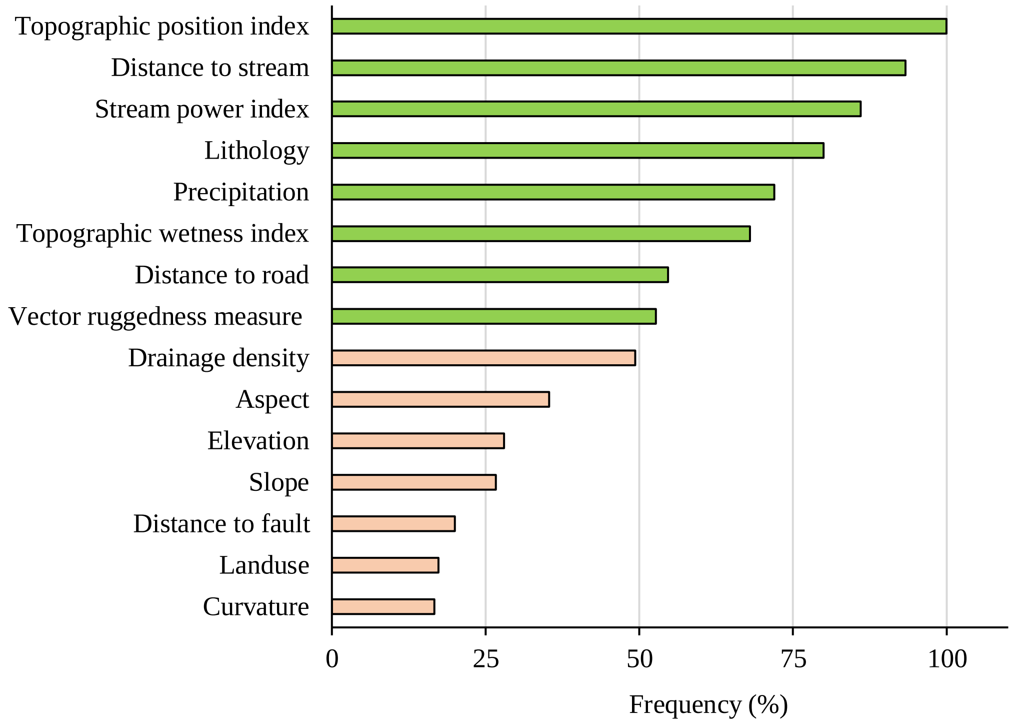

Figure S2. The occurrence frequency of the features in the recursive feature elimination (RFE) runs.
